# A Real-World Study of Patients with FGFR-Altered Advanced Solid Tumors after Exhaustion of Standard-of-Care Treatment

**DOI:** 10.1158/2767-9764.CRC-26-0233

**Published:** 2026-10-20

**Authors:** Levon Demirdjian, Hussein Sweiti, Raymond Harvey, Kristopher Standish, Nicole Stone, Simona Baculea, Joel Greshock, Spyros Triantos

**Affiliations:** Johnson & Johnson (United States), Spring House, Pennsylvania.

## Abstract

Real-world outcome data for patients with FGFR gene alteration–positive (*FGFR*alt) advanced/metastatic solid tumors after standard-of-care (SOC) exhaustion are lacking. De-identified data from a custom-curated Flatiron Health Foundation Medicine Clinico–Genomic database were used in this retrospective, noninterventional, observational study. Eligible patients were ≥18 years of age with *FGFR*alt advanced/metastatic disease who had exhausted SOC treatment. Real-world overall survival (rwOS), progression-free survival (rwPFS), and treatment patterns were assessed. Primary analyses were weighted for tumor types in the RAGNAR clinical trial (NCT04083976) and accounted for delayed study entry. Data from 230 patients (*FGFR* fusions: 68.3%; *FGFR* mutations: 37%) across 18 tumor types were analyzed. In primary analyses, the median (95% confidence interval) rwOS was 183 (148–236) days, and the median rwPFS was 87 (63–196) days. The median rwOS ranged from 86 [41–not available (NA)] to 356 (299–NA) days across tumor types and was 161 (106–212) days for patients with *FGFR* fusions and 236 (82–538) days for patients with *FGFR* mutations. Most patients had received one (67.4%) or two (18.2%) lines of therapy/treatment blocks at SOC exhaustion. From first-line therapy to SOC exhaustion, most common treatments were chemotherapy (100%) and immunotherapy (17.4%), and from best supportive care after SOC exhaustion to death was chemotherapy (65.2%), immunotherapy (16.1%), and a clinical trial drug (15.5%). These real-world survival and treatment-pattern findings highlight the poor prognosis of and unmet medical needs for patients with *FGFR*alt advanced/metastatic solid tumors who have exhausted SOC treatment.

**Significance:**

Findings from this real-world study—that patients with *FGFR*alt advanced/metastatic solid tumors have poor prognosis after SOC therapy exhaustion—contextualize results from the RAGNAR trial and suggest improved outcomes with erdafitinib. The rarity and poor prognosis of *FGFR* alterations highlight why randomized controlled trials in this population may be infeasible.

## Introduction

FGFRs are a family of highly conserved and widely distributed transmembrane tyrosine kinase receptors that play critical roles in cellular proliferation, migration, differentiation, and survival (1–5). FGFR gene alterations (*FGFR*alt; e.g., gene fusions, amplifications, rearrangements, and mutations) can result in aberrant activation of the FGFR signaling pathway and of downstream cell signaling pathways, such as the MAPK, PI3K, and JAK–STAT pathways. Dysregulation of FGFR signaling pathways has been implicated in cancer development and progression and has been associated with unfavorable prognosis and treatment resistance.

The prevalence and type of *FGFR*alt (*FGFR*alt) vary by cancer type (3, 4, 6, 7). For example, the most common *FGFR*alt in colorectal cancers are *FGFR1* amplifications, which may be associated with aggressive clinical behavior and poor prognosis (8). Furthermore, the overall *FGFR2* mutation rate was low (3.3%) in an analysis of >10,000 tumor samples across >30 cancer types; however, the highest mutation frequency was observed for cholangiocarcinomas [19.4% (fusions, 13.9%; mutations, 5.6%); ref. 9]. *FGFR2* fusions are particularly common in cholangiocarcinomas, in which they may constitutively activate *FGFR2* and drive subsequent tumor growth and survival (6). Lastly, pathogenic activating *FGFR3* aberrations show a stage-dependent frequency, and activating mutations are most prevalent in low-grade urothelial carcinoma tumors (6, 10).

The prognostic significance of *FGFR*alt also varies by cancer type. For instance, FGFR2 expression and genetic aberrations were shown to be clinically associated with improved outcomes in several cancers (9), and *FGFR3* aberrations were shown to be significantly associated with better progression-free survival (PFS) and overall survival (OS) in a retrospective analysis of >1,500 patients (10). However, mixed results in urothelial carcinoma have been reported in other studies (11–14), and in one retrospective, observational, comparative cohort analysis, *FGFR*alt had no impact on the prognosis of patients with advanced solid tumors receiving standard-of-care (SOC) treatment (15). For cholangiocarcinomas, a trend for improved survival was observed in patients having an *FGFR*alt (16, 17). In general, and across tumor types, the prognoses of patients with *FGFR*alt tumors and the impact of *FGFR*alt on natural history of disease in patients with *FGFR*alt tumors is poorly understood.

Second-generation FGFR inhibitors such as pemigatinib (FGFR1/2/3 inhibitor), futibatinib (pan-FGFR inhibitor), and erdafitinib (pan-FGFR inhibitor) are approved by the US FDA and European Medicines Agency for the treatment of certain advanced cancers with *FGFR*alt (18–20). Pemigatinib is approved for the treatment of advanced cholangiocarcinoma with *FGFR2* fusions or rearrangements and for myeloid/lymphoid neoplasms with *FGFR1* rearrangements (18), and futibatinib is approved for the treatment of advanced cholangiocarcinoma with *FGFR2* fusions or rearrangements (19). Erdafitinib is the only oral FGFR inhibitor therapy approved for the treatment of adult patients with locally advanced or metastatic urothelial carcinoma with susceptible *FGFR3* genetic alterations/mutations whose disease has progressed on or after ≥1 line of prior systemic therapy (20, 21).

Erdafitinib approval was supported by findings from the randomized, controlled, open-label, multicenter, confirmatory, phase III THOR trial (NCT03390504), in which erdafitinib treatment of patients with metastatic or unresectable urothelial carcinoma significantly prolonged OS, improved PFS, and increased the overall response rate (ORR) compared with chemotherapy (22). In the international, single-treatment arm, phase II RAGNAR trial (NCT04083976), erdafitinib was evaluated in patients with *FGFR*alt advanced solid tumors of any histology (except urothelial cancer) who were treated previously with ≥1 line of therapy (LOT) and had exhausted SOC treatment (23). In a pooled analysis of the broad-panel cohort (BPC), the ORR was 30% [95% confidence interval (CI), 24–36], median OS was 18.1 (13.40–24.28) months, and median PFS was 8.5 (6.83–9.72) months, confirming the efficacy of erdafitinib in diverse tumor types with prespecified *FGFR*alt (23). The single-arm design of the study, however, makes these outcomes challenging to contextualize.

To better characterize the real-world treatment landscape and outcomes of *FGFR*alt disease, assess the unmet medical need in patients with *FGFR*alt advanced solid tumors, and help contextualize the results of RAGNAR, we assessed real-world OS (rwOS) in patients with *FGFR*alt advanced solid tumors treated with best supportive care (BSC) after exhausting SOC; number and LOT after exhausting SOC, across tumor types and by individual tumor type; and real-world PFS (rwPFS) after exhausting SOC, across all tumor types.

## Materials and Methods

### Data source

Data were extracted from a custom-curated Flatiron Health (FH)–Foundation Medicine, Inc. (FMI) Clinico–genomic database (CGDB). In the FH-FMI CGDB, de-identified comprehensive, research-grade specimen, genomic, and other biomarker data from FMI are directly linked to de-identified, longitudinal clinical, treatment, and real-world outcomes from FH. Clinical data, including information about patients’ treatment, real-world outcomes, disease-specific biomarkers, and prognostic factors, were sourced from the electronic health records (EHR) of >5 million patients from >280 US community cancer centers (75%) and academic medical centers (25%). Longitudinal, patient-level, real-world clinical data were primarily sourced from EHRs, which may have been supplemented by other data (e.g., obituary and Social Security Death Index data), and included structured (preidentified; e.g., sex and birth date) and unstructured (free text; e.g., clinician notes) data. The FH-FMI CGDB includes two subsets, the disease-specific CGDB and the enhanced pan-tumor CGDB. If a patient did not satisfy disease-specific CGDB inclusion criteria, the patient became eligible for the enhanced pan-tumor CGDB. With this hierarchy, a patient could not exist in both subsets. Patients in a disease-specific CGDB have LOT data (i.e., disease specific), whereas patients in the pan-tumor CGDB have treatment block (TB) data (i.e., disease agnostic).

### Study design

**Figure 1.**
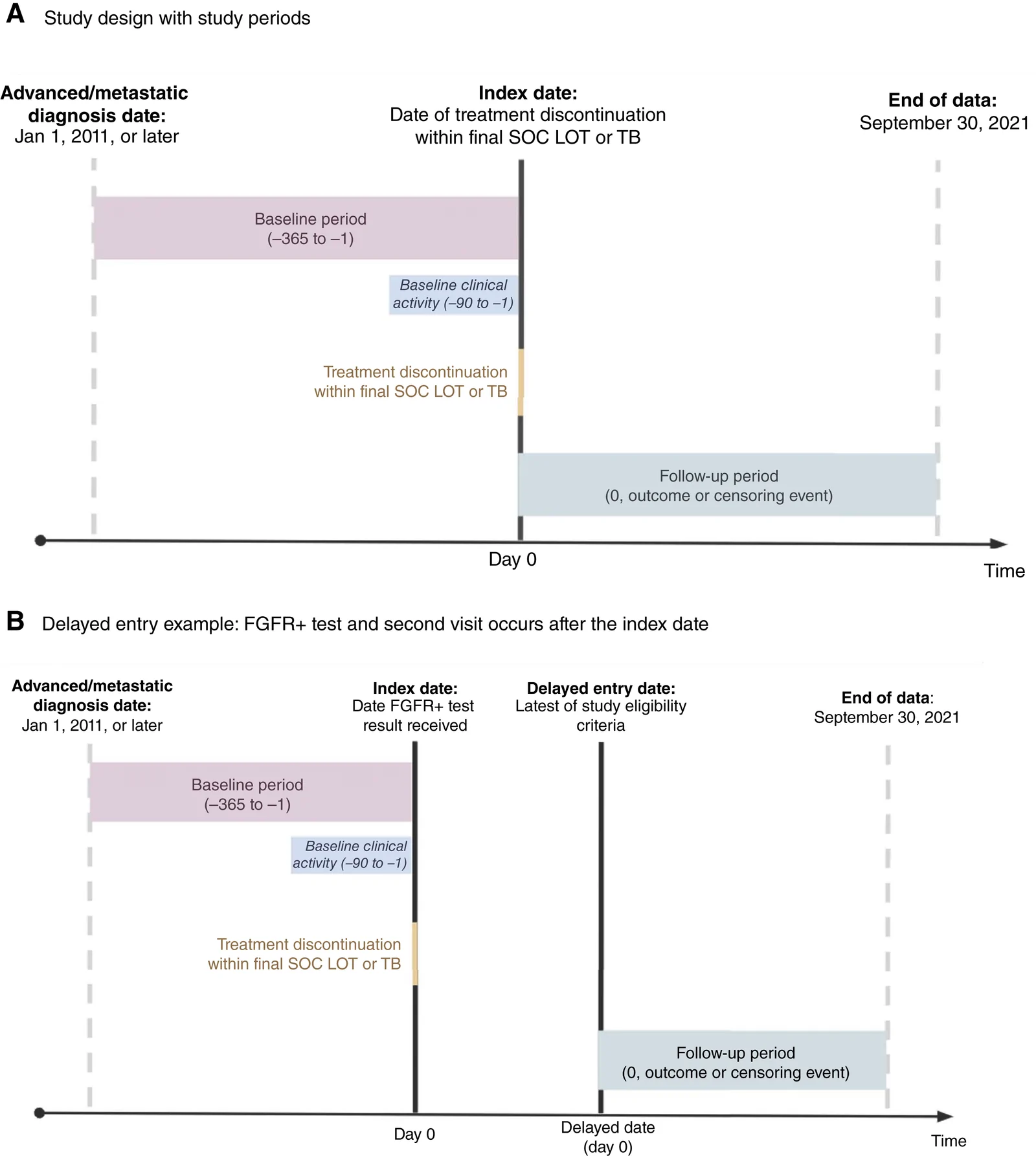
Study design with study periods (**A**) and primary analytic approach for survival incorporating delayed entry (**B**). The baseline period began 12 months before the index date and ended 1 day before the index date. The index date was defined as the date of treatment discontinuation within the final SOC LOT (disease-specific CGDB) or TB (pan-tumor CGDB), prespecified by tumor type for rwOS and date of salvage therapy initiation for rwPFS. If the date of discontinuation was missing, the date of last treatment use (e.g., administration, order, and oral) within the final SOC LOT or TB was used.

This retrospective, noninterventional, observational, cohort study involved secondary analysis of data collected during routine medical care. The study period (January 2011–September 2021) comprised a baseline period, index date (date of treatment discontinuation within the final SOC LOT or TB), and follow-up period, which lasted from the index date until the study outcome or censoring event, whichever came first (Fig. 1A). For rwOS, patients were followed from the index date until death or a censoring event (end of available data, date of last structured clinical activity, use of a selective FGFR inhibitor, last abstracted treatment episode where available, or last FMI report). In cases in which death occurred, death represented the end of the follow-up period. For rwPFS, patients were followed from the index date until progression/death, end of available data, use of a selective FGFR inhibitor, last clinic note date, or a 90-day gap without structured clinical activity. In cases in which death occurred after last clinical activity or last clinic note date, the first of these events represented the end of the follow-up period. BSC was defined as any form of disease management [e.g., pain medications, systemic anticancer salvage therapy (i.e., any use of antineoplastic cancer therapies after the index date, excluding radiotherapy and surgery), or no treatment] after the index date. Baseline clinical activity was defined as evidence of clinical activity (i.e., last clinic visit, last recording of vital information, a medication administration, or laboratory test/result report) within 90 days before the index date. Requiring baseline clinical activity ensured that patients were actively receiving care at a data-contributing provider before the index date for observability. The Sterling Institutional Review Board determined that this study was exempt from review pursuant to the terms of the US Department of Health and Human Service’s Policy for Protection of Human Research Subjects under 45 C.F.R. §46.104(d).

### Patients

For their data to be included in this study, patients had to be ≥18 years of age when diagnosed with advanced/metastatic disease (on or after January 1, 2011), had to have an *FGFR*alt in ≥1 solid tumor specimen at any time, and have evidence of SOC exhaustion in any LOT or TB. *FGFR*alt was defined as having ≥1 of 81 prespecified potential *FGFR* driver mutations or any *FGFR* fusion involving an intact kinase domain or an *FGFR* rearrangement with a named partner gene that was not *FGFR* and had a functional status of known or likely (i.e., the same mutations/fusions analyzed in RAGNAR). Criteria for SOC exhaustion were tumor-type specific and prespecified using clinical rationale prior to data collection to reflect US clinical practice guidelines at the time of study conduct (Supplementary Table S1). To contextualize real-world data with RAGNAR clinical trial data, only data for patients with tumor types observed in RAGNAR were included (Supplementary Table S2). Key exclusion criteria were *FGFR* resistance mutations (Supplementary Table S3) at any time; multiple primary tumor types, a bladder or hematologic malignancy, or central nervous system metastases within 5 years of the index date; receipt of erdafitinib, pemigatinib, infigratinib, futibatinib, or rogaratinib at any time before and including the index date; or a tumor type that was not included in RAGNAR. Exclusion criteria for analysis of rwPFS were no evidence of salvage therapy, a >90-day gap in activity between SOC exhaustion date and initiation of salvage therapy, receipt of an FGFR inhibitor between SOC exhaustion date and initiation of salvage therapy, a final clinic note date that occurred before initiation of salvage therapy, or a tumor type not assessed for progression by FH. The RAGNAR dataset included the BPC (primary; *n* = 217; ref. 23) and a cholangiocarcinoma expansion cohort (*n* = 35).

### Outcomes

rwOS and rwPFS were the primary clinical outcomes. Additional outcomes were types of systemic therapy used, in the advanced/metastatic setting after SOC exhaustion and to death for BSC; number of LOT and TBs in the advanced/metastatic setting before SOC exhaustion; and number of days from the index date to initiation of salvage therapy. An exploratory outcome was rwPFS after exhausting SOC.

### Analyses

This study was descriptive in nature, without hypothesis testing; therefore, no minimum sample size was required and statistical testing was not performed. Analyses were conducted using the Aetion Evidence Platform and RStudio version 4.1.2. Continuous variables were summarized using means (± SD) and medians (IQR), whereas categorical variables were summarized using frequencies, *n* (%).

rwOS and rwPFS were assessed across tumor types, and by individual tumor type, and were summarized using Kaplan–Meier curves, with 95% CI calculated using the log of the equal precision approach (24). The rwPFS analysis was considered exploratory because progression assessment timing and frequency in real-world clinical practice can vary by provider and by patient symptoms and was not documented for all tumor types in the databases, whereas progression assessment happens at regular intervals in clinical trials. Also, not all patients were assessed for progression. For example, patients receiving supportive care, and not salvage therapy, were not expected to be assessed for progression. Inclusion of these patients in the PFS analysis would have biased the outcomes.

The median time to event with 95% CIs was calculated using the Kaplan–Meier estimator, in which median time was estimable. Analyses of systemic therapy types were conducted using data from patients in the disease-specific CGDB only; data from the pan-tumor CGDB were not used because of the exploratory nature of TBs in the FH data.

The primary analytic approach for survival incorporated delayed entry (Fig. 1B) because some patients met inclusion criteria after the index date and began follow-up on the latest day on which they met all eligibility criteria. Delayed entry models (25) were used to mitigate immortal time bias, which could have arisen because patients included in the analysis needed to survive long enough to meet all study eligibility criteria and to have had ≥2 visits. During this period, the patients could not have died (hence, “immortal”). Delayed entry ensured that patients had adequate baseline clinical activity and data capture before cohort inclusion, thereby improving observability of baseline characteristics and alignment with study eligibility criteria. In a sensitivity “landmark” analysis—used to estimate survival probabilities in the absence of independent delayed entry (26)—patients with an outcome or censoring event between the index date and day 30 were excluded from analysis, accounting for a left-truncated dataset without requiring the assumption of conditional independence between entry and survival time required for delayed entry modeling to be valid. In this approach, patients began follow-up on day 30 after their index date. Patients who experienced the event of interest or were lost to follow-up before sequencing were not captured in the analytic cohorts. In the delayed entry analysis, conclusions cannot be drawn about patient survival before sequencing.

This real-world study reflects the distribution of tumor types present in the FH-FMI CGDB among patients who underwent *FGFR* testing and who have *FGFR*alt tumors. For the RAGNAR BPC, enrollment by tumor type was capped at approximately 30 patients to ensure that no single histology would dominate the study population. Accordingly, the real-world dataset was weighted to approximate the tumor type distribution in the total RAGNAR population (BPC + cholangiocarcinoma expansion cohort). For weighting, observations of overlap in tumor type and sample size ≥5 in both datasets were retained, but any tumor type with <5 patients in either dataset were excluded. Each observation in the datasets was given a weight representing the expected (target) population over the observed proportion of patients in each stratum. CIs for weighted Kaplan–Meier results were calculated via bootstrap or an alternative approach (e.g., robust variance estimation) as appropriate. An analysis without weighting, which included all eligible patients across all tumor types, was conducted to evaluate how sensitive the primary analysis results were to the choice of weights used.

The Eastern Cooperative Oncology Group performance status (ECOG-PS) score is used widely in oncology clinical trials but is less commonly collected in real-world databases. Moreover, the oncology EHR data included in the real-world dataset were not created primarily for research purposes, so information on certain variables such as ECOG-PS score may have been missing. High rates of missingness in ECOG-PS are a well-recognized limitation of oncology real-world data, in which performance status is often incompletely documented or captured in unstructured clinical records (27, 28). Therefore, no ECOG-PS score restriction was implemented for the real-world dataset. Patients for whom the most recent ECOG-PS score was not 0 or 1 within 90 days before the index date were excluded in a sensitivity analysis to evaluate outcomes in a subgroup more closely aligned with clinical trial populations (e.g., RAGNAR) to support contextualization of results, recognizing that such populations may differ systematically from real-world cohorts.

Planned stratifications of disease-specific CGDBs (e.g., type of tumor and *FGFR*alt type) were applied to group sizes >10 and analyzed using the primary analytic approach. *Post hoc* analyses included evaluation of rwPFS stratified by *FGFR*alt type (fusion vs. mutation) among patients with cholangiocarcinoma, the largest tumor type subgroup.

## Results

### Patients

Data for 230 (disease-specific CGDB, *n* = 161; pan-tumor CGDB, *n* = 69) patients across 18 tumor types were included in the analysis population dataset, which was used for all analyses except rwPFS (Supplementary Fig. S1). The rwPFS dataset comprised data for 112 patients. Mean (SD) age of patients was 60.7 (11.9) years; 62.2% were female; and 56.5% had an ECOG-PS score of 0 or 1 (Table 1). Most common metastatic sites were distant lymph nodes (33%), lung (30%), liver (27.4%), bone (22.6%), and peritoneum (17.8%). Overall, 163 (67.4%), 42 (18.2%), 11 (4.8%), 7 (3%), 5 (2.2%), and 1 (0.4%) patient had received 1, 2, 3, 4, 5, and 6 LOT/TBs at SOC exhaustion, respectively.

**Table 1.** Baseline patient demographics and clinical characteristics.

Characteristic	Real-world dataset	RAGNAR dataset		
	*N* = 230	BPC, *n* = 217 (23)	CCA, *n* = 35	Total, *n* = 252
Age at index date, years		​	​	​
Mean (SD)	60.7 (11.9)	55.6 (13.5)	57 (9.9)	55.8 (13.1)
Median (IQR)	63 (54–69.3)	57	57	57
Range	27–84	12–79	40–74	12–79
≥12 years to <18 years, *n* (%)	0 (0)	2 (0.9)	0 (0)	2 (0.8)
18 to <65 years of age, *n* (%)	132 (57.4)	162 (74.7)	25 (71.4)	187 (74.2)
≥65 years of age, *n* (%)	98 (42.6)	53 (24.4)	10 (28.6)	63 (25)
Sex, *n* (%)		​	​	​
Female	143 (62.2)	97 (44.7)	23 (65.7)	120 (47.6)
Male	87 (37.8)	120 (55.3)	12 (34.3)	132 (52.4)
Race/ethnicity, *n* (%)		​	​	​
Asian	5 (2.2)	57 (26.3)	11 (31.4)	68 (27)
Black or African American	23 (10)	6 (2.8)	0 (0)	6 (2.4)
White	164 (71.3)	112 (51.6)	18 (51.4)	130 (51.6)
Hispanic or Latino	2 (0.9)	9 (4.1)	1 (2.9)	10 (4)
Other	25 (10.9)	4 (1.8%)	0 (0)	4 (1.6)
Unknown, race + ethnicity	11 (4.8)	—	—	—
Unknown, race/ethnicity	—	38 (17.5)/35 (16.1)	6 (17.1)/7 (20)	46 (18.3)/42 (16.7)
Country, *n* (%)		​	​	​
United States	230 (100)	48 (22.1)	3 (8.6)	51 (20.2)
Argentina	—	2 (0.9)	0 (0)	2 (0.8)
Australia	—	12 (5.5)	2 (5.7)	14 (5.6)
Belgium	—	10 (4.6)	5 (14.3)	15 (6)
Brazil	—	8 (3.7)	1 (2.9)	9 (3.6)
China	—	12 (5.5)	3 (8.6)	15 (6)
France	—	26 (12)	5 (14.3)	31 (12.3)
Germany	—	22 (10.1)	4 (11.4)	26 (10.3)
Italy	—	3 (1.4)	0 (0)	3 (1.2)
Japan	—	23 (10.6)	3 (8.6)	26 (10.3)
Poland	—	3 (1.4)	0 (0)	3 (1.2)
South Korea	—	6 (2.8)	1 (2.9)	7 (2.8)
Spain	—	16 (7.4)	3 (8.6)	19 (7.5)
Taiwan	—	12 (5.5)	4 (11.4)	16 (6.3)
United Kingdom	—	14 (6.5)	1 (2.9)	15 (6)
ECOG-PS score, *n* (%)		*n* = 215 (adults)	*n* = 35	*n* = 250 (adults)
0	50 (21.7)	65 (30.2)	16 (45.7)	81 (32.4)
1	80 (34.8)	149 (68.7)	19 (54.3)	168 (67.2)
2	32 (13.9)	1 (<0.01)	0 (0)	1 (0.4)
3	4 (1.7)	0 (0)	0 (0)	0 (0)
4	0 (0)	0 (0)	0 (0)	0 (0)
Missing	64 (27.8)	0 (0)	0 (0)	0 (0)
Metastases, *n* (%)		*n* = 179	*n* = 33	*n* = 212
0	53 (23)	0 (0)	0 (0)	0 (0)
1	80 (34.8)	40 (22.3)	7 (21.2)	47 (22.2)
2	58 (25.2)	50 (27.9)	11 (33.3)	61 (28.8)
3 (real world) and ≥3 (RAGNAR)	22 (9.6)	89 (49.7)	15 (45.5)	104 (49.1)
4	10 (4.3)			
5	5 (2.2)			
6	1 (0.4)			
7	1 (0.4)			
*FGFR*alt type, *n* (%)^a^		​	​	​
Fusion	157 (68.3)	144 (66.4)	32 (91.4)	176 (69.8)
Mutation	85 (37)	75 (34.6)	3 (8.6)	78 (31)
Comorbidities, *n* (%)		​		
Cerebrovascular disease	14 (6.1)	​		
Chronic pulmonary disease	46 (20)			
Congestive heart failure	14 (6.1)			
Connective tissue disease	8 (3.5)			
Diabetes mellitus with end organ damage	8 (3.5)			
Diabetes mellitus without end organ damage	51 (22.2)			
Hemiplegia/paraplegia	3 (1.3)			
Mild chronic liver disease	30 (13)			
Moderate to severe renal disease	18 (7.8)			
Myocardial infarction	12 (5.2)			
Peptic ulcer disease	16 (7)			
Peripheral vascular disease	17 (7.4)			
Group stage at initial diagnosis, *n* (%)				
0	18 (7.8)			
1	15 (6.5)			
2	27 (11.7)			
3	133 (57.8)			
4	37 (16.1)			
Unknown/not populated	18 (7.8)			
Tumor grade at initial diagnosis, *n* (%)				
Grade 1	3 (1.3)			
Grade 2	9 (3.9)			
Grade 3	7 (3)			
Unknown	211 (91.7)			
Blood: TMB version 1^b^				
Mean (SD)	7.33 (4.93)			
Median (IQR)	7.59 (3.16–11.38)			
Missing, *n* (%)	225 (97.8)			
Blood: TMB version 2^c^				
Mean (SD)	8.09 (3.64)			
Median (IQR)	7.59 (5.06–11.38)			
Missing, *n* (%)	225 (97.8)			
Tissue: TMB version 1^b^				
Mean (SD)	4.3 (8.5)			
Median (IQR)	2.1 (0.89–5)			
Missing, *n* (%)	124 (53.9)			
Tissue: TMB version 2^c^				
Mean (SD)	5.1 (8.7)			
Median (IQR)	2.6 (1.3–6.1)			
Missing, *n* (%)	143 (62.2)			
Visceral metastases, *n* (%)^d^				
Mean (SD)	0.64 (0.7)			
Median (IQR) for RWE	1 (0–1)			
0	113 (49.1)			
1	93 (40.4)			
2	18 (7.8)			
3	6 (2.6)			

Abbreviations: CCA, cholangiocarcinoma; NA, not available; RWE, real-world evidence; TMB, tumor mutation burden.

a Not mutually exclusive; patients could have had evidence of both a fusion and a mutation alteration type or multiple FGFR mutations.

b A research use–only tissue TMB value generated retrospectively for all tissue specimens, including those for which a tissue TMB score was not included as part of the physician-facing test report, using latest version of FMI’s research use–only tissue TMB algorithm and current reference annotations with respect to each variant’s functional status and likelihood of being somatic vs. germline. This score generally approximates the tissue TMB score that would result from running all specimens on the same version of the analysis pipeline.

c The tissue TMB value (reported as a float value, indicating the number of nondriver somatic coding mutations detected per MB of genome sequenced) as calculated by the analysis pipeline at the time of testing for a tissue specimen. Curation of scores generated by the analysis pipeline occasionally occurred before completion of the physician-facing report and was not captured in the score.

d Includes metastases to the adrenal glands, kidney, liver, lung, ovary, pancreas, and spleen.

Proportions of patients with each tumor type within each dataset, and consequent weighting, are shown in Table 2. Overall, 68.3% of patients had *FGFR* fusions and 37% of patients had *FGFR* mutations (Table 1); breast cancer, 70.6% vs. 29.4%; cholangiocarcinoma, 84.5% vs. 20.2%; endometrial, 8.3% vs. 91.7%; gastric esophageal and gastroesophageal, 75% vs. 33.3%; glioma, 90% vs. 15%; and non–small cell lung cancer, 50% vs. 55%. Most common *FGFR*alt were FGFR3–TACC3 [45 (19.6%)], followed by FGFR2–BICC1 [30 (13%)].

**Table 2.** Frequency of tumor types and weighting for analysis.

​	Real-world dataset primary analysis cohort, *n* = 230		RAGNAR dataset, *n* = 252 (BPC and cholangiocarcinoma expansion cohorts)		Weight
	Proportion	*n*	Proportion	*n*	
Tumor types used for weighting					
Cholangiocarcinoma	0.37	84	0.26	66	0.717
Pan-tumor unknown	0.10	22	0.03	8	0.332
Glioma	0.09	20	0.14	37	1.597
Non–small cell lung cancer	0.09	20	0.09	23	1.141
Breast	0.07	17	0.06	16	0.859
Endometrial	0.05	12	0.03	8	0.608
Gastroesophageal junction	0.05	12	0.06	16	1.217
Pan-tumor cervical	0.04	10	0.02	6	0.548
Pancreatic	0.02	5	0.07	18	3.286
Pan-tumor anal	0.02	5	<0.01	1	0.183
Total	—	207	—	199	—
Tumor types not used for weighting					
Pan-tumor gallbladder	<0.02	4	<0.01	1	—
Pan-tumor duodenal	<0.01	1	<0.01	1	—
Colorectal	<0.02	<5	0.03	8	—
Head and neck	<0.02	<5	0.06	15	—
Pan-tumor head and neck	<0.02	<5	0	0	—
Pan-tumor soft tissue	<0.02	<5	0.01	3	—
Prostate	<0.02	<5	<0.01	2	—
Ovarian	<0.02	<5	0.03	8	—
Other^a^	—	NA	—	15	—
Total	—	23	—	53	—

Abbreviation: NA, not applicable.

Weight = proportion in the RAGNAR dataset divided by the proportion in the real-word dataset. Weighted to approximate distributions in the RAGNAR trial and includes real-world patients who met inclusion criteria after the index date and began follow-up on the latest day on which they met all eligibility criteria. Tumor types with <5 patients in the FH dataset were not included in weighting.

Bladder and duodenal tumors were combined within the FH dataset; these tumor types were not included in weighting because <5 patients had each tumor type.

a Other includes RAGNAR tumor types not represented in or with <5 patients with the tumor type in the FH dataset = adenoid cystic carcinoma (1), basal cell carcinoma (1), conjunctival epidermoid carcinoma (1), gastrointestinal stromal tumor (1), germ cell tumor (1), mesothelioma (1), parathyroid carcinoma (1), salivary gland cancer (5), testicular cancer (1), thymic cancer (1), and thyroid carcinoma (1).

### rwOS

**Figure 2.**
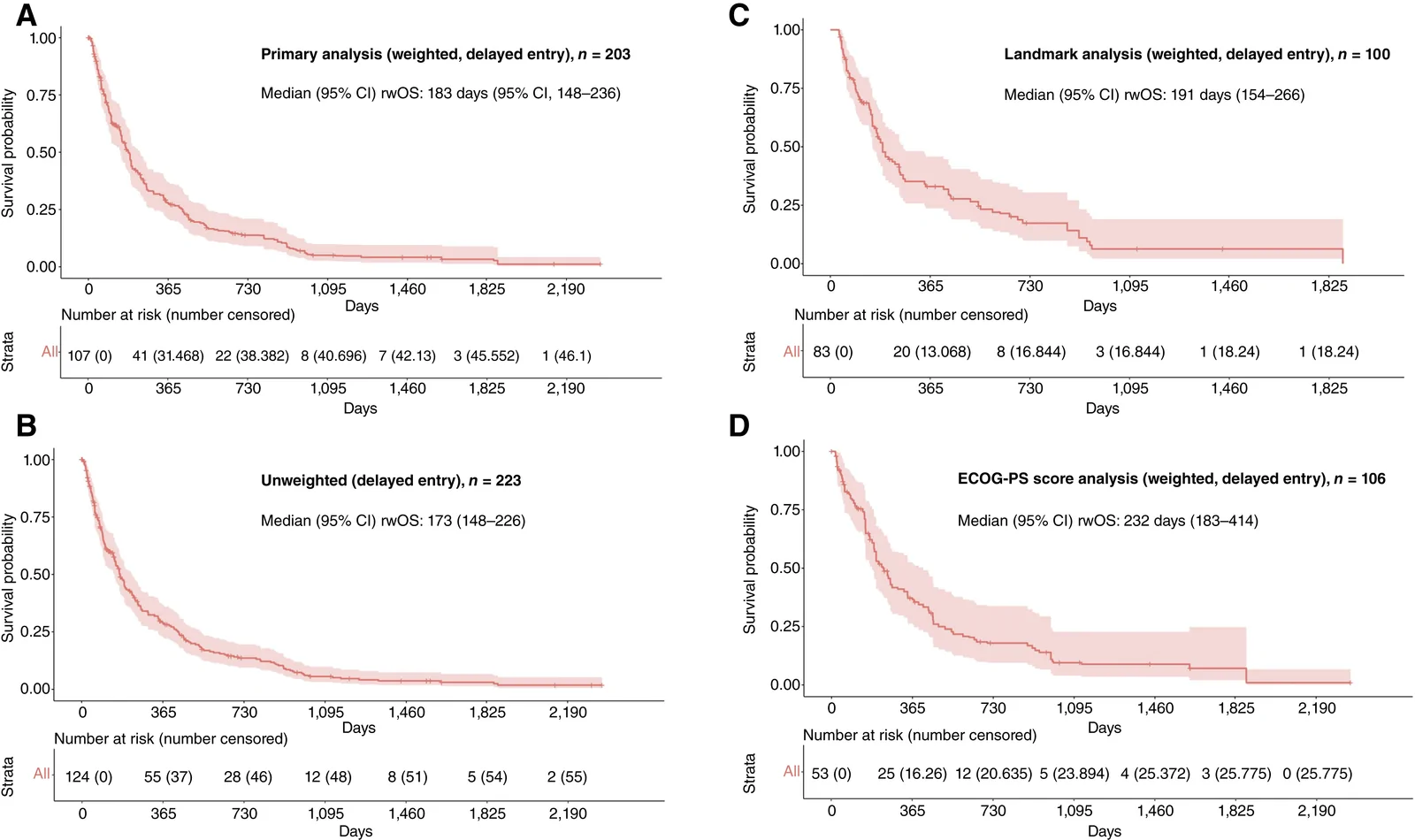
rwOS in the primary analysis (**A**), unweighted sensitivity analysis (**B**), sensitivity landmark analysis (**C**), and ECOG-PS score analysis (**D**). Primary analysis (weighted, delayed): weighted to approximate distributions in the RAGNAR trial and includes patients who met inclusion criteria after the index date and began follow-up on the latest day on which they met all eligibility criteria; tumor types with <5 patients in either dataset were not included in weighting. Unweighted analysis: not weighted to approximate distributions in the RAGNAR trial. Landmark analysis: patients with an outcome or censoring event between the index date and day 30 were excluded from analysis, accounting for a left-truncated dataset without requiring the assumption of conditional independence between entry and survival time. ECOG-PS score analysis: patients for whom the most recent ECOG-PS score was not 0 or 1 within 90 days before index date were excluded. Sample sizes vary because some patients were dropped in the time-to-event analysis because of weighting (tumor types with <5 patients in either group were removed), death or censoring at or before the index date, and/or a delayed entry date after a censoring event. Because delayed entry modeling was used, patients entered the risk sets at the delayed entry date; therefore, the number at risk at time 0 may not match with the total *n*, reflecting the fact that some patients had not yet entered the risk set at time 0. Delayed entry, patients who met inclusion criteria after the index date and began follow-up on the latest day on which they met all eligibility criteria; weighted, the real-world dataset was weighted to approximate distributions in the RAGNAR trial (target population).

The median rwOS was 183 (95% CI, 148–236; IQR, 75–432) days across tumor types in the primary analysis (*n* = 203; Fig. 2A). When stratified by tumor type, the median rwOS ranged from 86 [95% CI, 41–not available (NA)] days for patients with gastric and esophageal cancer (*n* = 20) to 365 (95% CI, 299–NA) days for patients with endometrial cancer (*n* = 12; Supplementary Fig. S2). When stratified by *FGFR*alt type at entry, the median rwOS was 161 (95% CI, 106–212) days for patients with *FGFR* fusions (*n* = 152) and 236 (95% CI, 149–372) days for patients with *FGFR* mutations (*n* = 82; Supplementary Fig. S3).

In a sensitivity analysis without weighting (*n* = 223), the median rwOS was 173 (95% CI, 148–226; IQR, 70–437) days (Fig. 2B). The median rwOS was 191 (95% CI, 154–266) days in the sensitivity landmark analysis (Fig. 2C) and 232 (95% CI, 183–414) days when patients for whom the most recent ECOG-PS score was not 0 or 1 within 90 days before the index date were excluded from analysis (Fig. 2D).

### Real-world treatment patterns

Among the 161 patients in the disease-specific CGDB, all received chemotherapy, 17.4% received immunotherapy, <10% received hormonal therapy or a clinical trial drug, and <1.5% received an antibody–drug conjugate or an αβ emitter from first-line therapy in the advanced/metastatic setting to SOC exhaustion (Table 3). Biologic and nonbiologic targeted therapies were administered to 11.8% and 6.8% of patients, respectively. From BSC to death, 65.2% of patients were treated with chemotherapy, 16.1% with immunotherapy, 15.5% with a clinical trial drug, and <6% with hormonal therapy or an antibody-drug conjugate; none were treated with an αβ emitter. Biologic and nonbiologic targeted therapies were administered to 9.3% and 21.7% of patients, respectively. Among 182 patients who received salvage therapy, the median number of days from the index date to initiation of salvage therapy was 25 (range, 10.50–50.25) days. Treatment patterns by individual tumor type are shown in Supplementary Table S4.

**Table 3.** Real-world systemic therapy patterns.

Number of LOT in the metastatic/advanced setting before SOC exhaustion (disease-specific CGDB, *n* = 161^a^)	
Mean (SD)	1.7 (1.1)
Median (IQR)	1 (1–2)
Number of TBs in the metastatic/advanced setting before SOC exhaustion (pan-tumor CGDB, *n* = 69)	
Mean (SD)	1.1 (0.2)
Median (IQR)	1 (1–1)
Number of days from the index date to initiation of salvage therapy (*n* = 182)	
Mean (SD)	61.7 (124.1)
Median (IQR)	25 (10.5–50.3)

Types of treatment following SOC therapy (disease-specific CGDB, *n* = 161^a^)		
​	First-line therapy in the advanced/metastatic setting to SOC exhaustion, *n* (%)^b^	BSC to death, *n* (%)^b^
αβ emitter	1 (0.6)	0 (0)
Antibody–drug conjugate	2 (1.2)	3 (1.9)
Chemotherapy	161 (100)	105 (65.2)
Clinical study drug	9 (5.6)	25 (15.5)
Hormonal therapy	15 (9.3)	9 (5.6)
Immunotherapy	28 (17.4)	26 (16.1)
Targeted/biologic^c^	19 (11.8)	15 (9.3)
Targeted/nonbiologic^d^	11 (6.8)	35 (21.7)

a Includes only the disease-specific CGDB because of the exploratory nature of TBs in FH data.

b Not mutually exclusive; patients could have been exposed to multiple therapy types, either within the same line or across multiple lines.

c Bevacizumab, bevacizumab-awwb, bevacizumab-bvzr, cetuximab, olaratumab, panitumumab, pertuzumab, ramucirumab, trastuzumab, trastuzumab-anns, or trastuzumab-dkst.

d Abemaciclib, afatinib, alpelisib, avapritinib, dabrafenib, dasatinib, erdafitinib, erlotinib, everolimus, lapatinib, lenvatinib, olaparib, osimertinib, palbociclib, pazopanib, pemigatinib, regorafenib, ripretinib, sorafenib, sunitinib, or temsirolimus.

### rwPFS

**Figure 3.**
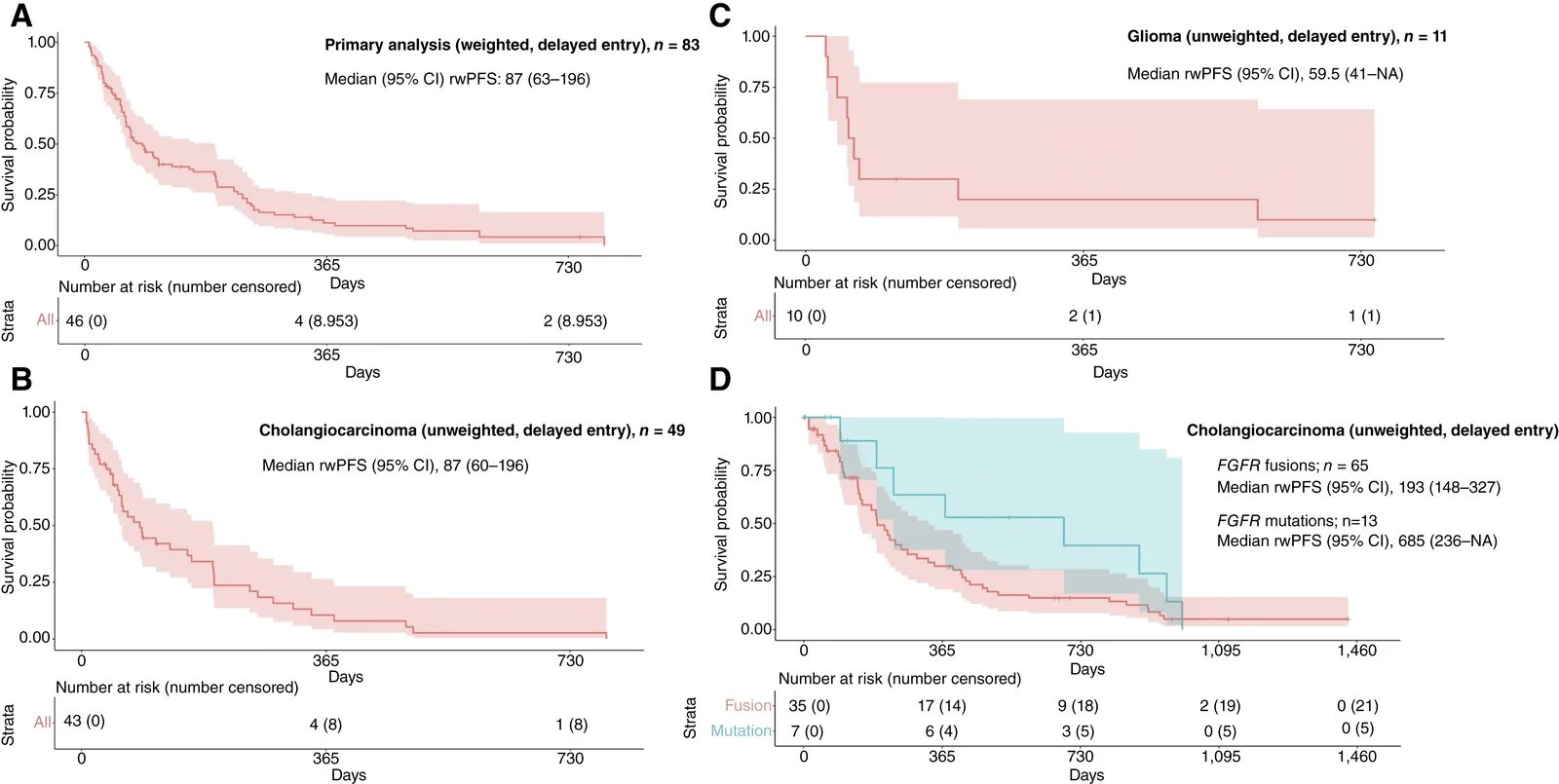
rwPFS in the primary analysis (**A**) and unweighted sensitivity analyses stratified by tumor type (**B–D**). Primary analysis (weighted, delayed): weighted to approximate distributions in the RAGNAR trial and includes patients who met inclusion criteria after the index date and began follow-up on the latest day on which they met all eligibility criteria; tumor types with <5 patients in either dataset were not included in weighting. Unweighted analysis: not weighted to approximate distributions in the RAGNAR trial. Landmark analysis: patients with an outcome or censoring event between the index date and day 30 were excluded from analysis, accounting for a left-truncated dataset without requiring the assumption of conditional independence between entry and survival time. ECOG-PS score analysis: patients for whom the most recent ECOG-PS score was not 0 or 1 within 90 days before index date were excluded.

The median rwPFS was 87 (95% CI, 63–196; IQR, 43–238) days in a primary analysis (weighted, delayed entry; *n* = 83; Fig. 3A). When stratified by tumor type in sensitivity analyses (unweighted, delayed entry), rwPFS was 87 (95% CI, 60–196; IQR, 37–198) days for cholangiocarcinoma (*n* = 49; Fig. 3B) and 59.5 (95% CI, 41–NA; IQR, 41–200) days for glioma (*n* = 11; Fig. 3C). When these tumor types were further stratified by *FGFR*alt type in *post hoc* analyses (unweighted, delayed entry), the median rwPFS was 193 (95% CI, 148–327) days for *FGFR* fusions (*n* = 65) and 685 (95% CI, 236–NA) days for *FGFR* mutations (*n* = 13; Fig. 3D).

## Discussion

The rarity of *FGFR*alt across most nonurothelial cancers supports tumor-agnostic study designs. Findings from this real-world study of treatment patterns and outcomes among 230 patients with *FGFR*alt advanced/metastatic solid tumors who had exhausted SOC treatment highlight the poor prognosis of and the unmet medical need for improved therapies for these patients. Furthermore, contextualizing outcomes with those from the RAGNAR clinical trial (23) suggests improved outcomes in patients receiving the selective FGFR inhibitor, erdafitinib, for which the median OS was 18.1 months. The observed poor outcomes highlight the challenges in randomizing a patient population that has exhausted all SOC. Previously, we evaluated the prognostic relevance of *FGFR*alt within real-world datasets, including analyses comparing outcomes among patients with and without *FGFR*alt across tumor types (15). Here, we focused on characterizing the natural history of *FGFR*alt disease in the post–SOC exhaustion setting. *FGFR*alt in specific disease contexts, including in high-risk non–muscle-invasive bladder cancer (29), have been conducted using real-world CGDBs. In at least one analysis, alternative approaches to identify patients who may benefit from FGFR-targeted therapies were explored, further highlighting the potential role of real-world data in understanding FGFR biology and treatment responsiveness.

The use of real-world data in the present study was intended to complement prospective clinical trial findings from RAGNAR. Moreover, findings underscore the potential value of real-world evidence for contextualizing single-arm trial results in settings of substantial unmet need, particularly when prospective randomized studies may be challenging in rare biomarker-defined populations.

Overall, 68.3% and 37% of patients in the real-world study had *FGFR* fusions and *FGFR* mutations, respectively, which is similar to that observed in the RAGNAR BPC (fusions: 66%; mutations: 35%; ref. 23). In the real-world population, outcomes for patients with *FGFR* fusions were worse than for patients with *FGFR* mutations, overall (rwOS, 161 vs. 236 days) and among patients with cholangiocarcinoma (rwOS, 161 vs. 236 days; rwPFS, 193 vs. 685 days). In contrast, clinical trial outcomes for patients with *FGFR* fusions were similar to or better than those with *FGFR* mutations. In the phase II dose-escalation/dose-expansion FIGHT-101 trial of pemigatinib in molecularly unselected patients with advanced malignancies (*N* = 128), ORR was 25% for *FGFR* fusions/rearrangements (*n* = 20) and 23.1% for *FGFR* mutations (*n* = 13), and the median PFS was 5.7 and 5 months, respectively (30). In the RAGNAR clinical trial, ORR was 32.6% for *FGFR* fusions and 25.3% for *FGFR* mutations. Additionally, ORR among patients (*N* = 54) enrolled in FGFR-targeting phase I and/or phase II trials at a single UK institution was better for patients with *FGFR* rearrangements (38%) versus those with *FGFR* amplifications (25%) or mutations (25%; ref. 31). These findings should be interpreted with caution because of differences in patient populations and study conduct (e.g., study designs and timeframes, geography, tumor types, and types of *FGFR*alt).

Given the heterogeneity in the natural history and prognostic impact of *FGFR*alt across tumor types, aggregated OS estimates should be interpreted with caution. Tumor-specific outcomes were, therefore, reported. Substantial variability was observed across histologies. Methodologic approaches, including histology weighting and sensitivity analyses, were used to mitigate the impact of this heterogeneity, yielding consistent findings. The broad inclusion approach was necessary to capture *FGFR*-altered cases across rare malignancies and to align with the tumor-agnostic design of RAGNAR.

In the present real-world study, two-thirds of patients eligible for analysis had received one LOT/TB at SOC exhaustion, almost one in five patients had received two LOT/TBs, and one in 10 had received ≥3 LOT/TBs. All 161 patients in the disease-specific CGDB had received chemotherapy, and 17.4% and 11.8% of patients also received immunotherapy and biologics, respectively, from first-line therapy in the advanced/metastatic setting to SOC exhaustion. Fewer than one in 10 patients had received hormonal therapy, nonbiologics, or a clinical trial drug, respectively. Rarely, patients received an antibody–drug conjugate or an αβ emitter. From BSC to death, almost two-thirds of patients were treated with chemotherapy and approximately one in five were treated with a nonbiologic. Less common treatments were immunotherapy, a clinical trial drug, biologics, hormonal therapy, or an antibody-drug conjugate; none were treated with an αβ emitter. These findings reflect the various SOC regimens that were available for the tumor types in the patient population at the time of study conduct. The patterns of care after exhausting SOC suggest that the majority of patients were offered some type of salvage systemic therapy, most commonly chemotherapy. However, efficacy was limited and outcomes were poor, highlighting the unmet need for improved treatments after exhausting SOC, the limited benefit of salvage systemic therapy, and the importance of offering the option of novel agents in clinical trials to these patients.

Although more prevalent in certain tumor types (3, 4), *FGFR*alt generally are rare or unknown because of lack of testing. A strength of this real-world study was the opportunity to query large databases to identify a large number of patients for evaluation. The distribution of tumor types in this study is reflective of a real-world population of patients with advanced/metastatic, *FGFR*alt solid tumors.

In addition to strengths, some limitations of this real-world study must be acknowledged. Patients may have been lost to follow-up, and mortality may have been underestimated—despite use of external sources to enhance patient deaths. As with any real-world, EHR-derived data source, the FH-FMI CGDB is subject to limitations, including incomplete or missing capture of key clinical variables, variability in documentation practices across sites, and the potential for misclassification or measurement error in structured and unstructured data abstraction. The use of delayed entry may have influenced survival estimation by excluding early post–index date person-time for some patients, which could result in shorter OS estimates. In addition, findings from this real-world study were used to contextualize results from the RAGNAR BPC (23) because direct comparison of rwPFS and PFS was precluded by lower real-world frequency of progression assessments, possibly leading to artificially inflated rwPFS estimates. Although some patients’ baseline characteristics [e.g., proportion of *FGFR* fusions (69% each) and mutations (31% vs. 37%, respectively)] were comparable, other baseline characteristics (Table 1) and the conduction of the two studies (Supplementary Table S5) varied in some respect. Many of these differences, however, were accounted for in the real-world data analyses, were assessed in sensitivity analysis, or both.

In contrast to RAGNAR (23), in which patients had to have an ECOG-PS score of 0 (32%) or 1 (67%) for inclusion, patients in the primary real-world dataset were included regardless of baseline ECOG-PS score [0: 22%; ≥1: 50% (missing: 28%)]. When limiting the analysis to patients with baseline ECOG-PS scores of 0 to 1, which is a typical inclusion criterion in oncology clinical trials (27, 28), rwOS was 232 days, indicating that patients with poorer prognoses at baseline (ECOG-PS score ≥2) had worse outcomes. The type and distribution of tumor types included in RAGNAR and in the real-world study also differed. Some of the tumor types included in RAGNAR were not included in the FH-FMI CGDB and *vice versa*. To address this limitation, the real-world study included only tumor types in RAGNAR, and to address potential differences in tumor type distribution, weighting was used in the real-world data analyses to approximate tumor type distribution in RAGNAR. The median rwOS was 173 days in an unweighted sensitivity analysis (vs. 183 days in the primary, weighted analysis), suggesting that the primary analysis findings were not necessarily specific to the exact tumor type distribution seen in RAGNAR. Moreover, the index dates were defined differently in RAGNAR (date of initiation of erdafitinib) and in this real-world study (date of treatment discontinuation within the final SOC LOT or TB). Therefore, an additional period of time (i.e., “gap time”) existed between the index date and subsequent LOT/TB start date for patients in the real-world analysis. This gap time added time to real-world survival outcomes, potentially enhancing these outcomes. Furthermore, patients in RAGNAR were indexed on receipt of erdafitinib, whereas patients in the real-world study were indexed on the date of SOC exhaustion. Patients in RAGNAR elected to enroll in a clinical trial and lived long enough after SOC to meet trial entry criteria. Therefore, the number of SOC therapies and/or time to initiation of salvage therapy likely differed in the two populations. Finally, delayed entry modeling was incorporated into the real-world study. However, in a sensitivity analysis used to estimate survival probabilities in the absence of independent delayed entry, the median rwOS was 191 days, implying that the effects of potential violations to the delayed entry assumption in the primary analysis were minimal.

In summary, these real-world survival and treatment-pattern findings highlight the poor prognosis of patients with *FGFR*alt advanced/metastatic solid tumors who have exhausted SOC treatment and emphasize the unmet medical need for improved therapies for these patients. Contextualizing the real-world outcomes with those from the RAGNAR clinical trial suggests improved outcomes among trial patients receiving the selective FGFR inhibitor, erdafitinib. Moreover, the observed real-world outcomes after exhausting SOC may signify why randomized controlled trials may not be feasible in this patient population.

## Supplementary Material

Supplementary Figure S1 Figure S1. Real-world dataset patient disposition

Supplementary Figure S2 Figure S2. Real-world overall survival stratified by tumor type (unweighted, delayed entry sensitivity analysis)

Supplementary Figure S3 Figure S3. Real-world overall survival stratified by type of FGFR alteration (unweighted, delayed entry sensitivity analysis)

Supplementary Table S1 Criteria for standard-of-care exhaustion by tumor type

Supplementary Table S2 Table S2. Tumor types studied in the RAGNAR trial and real-world study

Supplementary Table S3 Table S3. Fibroblast growth factor receptor valine gatekeeper and resistance alterations

Supplementary Table S4 Table S4. Real-world systemic therapy patterns by tumor type

Supplementary Table S5 Table S5. Differences in study conduct between the real-world study and the RAGNAR clinical trial

## Data Availability

The data-sharing policy of Janssen Pharmaceutical Companies of Johnson & Johnson is available at https://www.janssen.com/clinical-trials/transparency. As noted on this site, requests for access to study data can be submitted through the Yale Open Data Access Project site at http://yoda.yale.edu.
