# Supplementary material for: A Real-World Study of Patients with FGFR-Altered Advanced Solid Tumors after Exhaustion of Standard-of-Care Treatment
Source: Cancer Res Commun. 2026 Oct 20;6(10):2451–62. doi: 10.1158/2767-9764.CRC-26-0233 (PMC13680822; doi:10.1158/2767-9764.CRC-26-0233)
Supplement: Supplementary file 1 [file crc-26-0233_supplementary_table_s1_suppst1.pdf]

Supplementary table 1: Criteria for standard-of-care exhaustion by tumor type[a]

Tumor type	SOC therapy exhaustion requirement(s)[b]
Disease-specific CGDB	
Breast cancer	Must have completed exposure to ≥2 LOT (single agent or in combination) in the locoregional or metastatic setting, with each LOT containing ≥1 of the following distinct from the therapy of interest in the other line: doxorubicin, liposomal doxorubicin, paclitaxel, docetaxel, capecitabine, gemcitabine, vinorelbine, ixabepilone, eribulin, paclitaxel protein-bound, carboplatin, cisplatin, epirubicin, cyclophosphamide, and sacituzumab govitecan
Cholangiocarcinoma	Must have completed exposure to ≥1 LOT with cisplatin + gemcitabine or oxaliplatin + fluorouracil
Colorectal cancer	Must have completed exposure to ≥1 LOT and have been exposed to all of the following: oxaliplatin, irinotecan, and fluorouracil or capecitabine in any order, combination, and number of LOT
Advanced endometrial cancer	Must have completed exposure to ≥1 LOT and have been exposed to all of the following: carboplatin or cisplatin and paclitaxel or docetaxel or doxorubicin or liposomal doxorubicin or nabpaclitaxel or ifosfamide in any order, combination, and number of LOT

Gastric/esophageal/gastroesophageal junction (esophageal)	Must have completed exposure to ≥1 LOT containing ≥1 of the following: complete exposure to a platinum, fluoropyrimidine, and/or taxane class; cisplatin, carboplatin, oxaliplatin, fluorouracil, capecitabine, paclitaxel, or docetaxel
Gastric/esophageal/gastroesophageal junction (gastric/gastroesophageal junction)	Must have completed exposure to ≥1 LOT in any order and number of LOT containing ≥1 of the following: ramucirumab and complete exposure to a platinum, fluoropyrimidine and/or taxane class): cisplatin, carboplatin, oxaliplatin, fluorouracil, capecitabine, paclitaxel or docetaxel
Squamous cell carcinoma of the head and neck	Must have completed exposure to ≥1 LOT containing at least pembrolizumab + carboplatin or cisplatin + fluorouracil, or ≥2 LOT in any order, combination, and number of LOT with one line containing carboplatin or cisplatin + docetaxel or paclitaxel or fluorouracil and one line containing pembrolizumab or nivolumab
Non-small cell lung cancer (non-squamous + lung, not otherwise specified)	Must have completed exposure to at least, in any order and number of LOT, 1 LOT with carboplatin or cisplatin) and pemetrexed or gemcitabine or docetaxel or paclitaxel or paclitaxel protein-bound, and a checkpoint inhibitor (pembrolizumab, nivolumab, atezolizumab or durvalumab) at any time, including before advanced diagnosis date
Non-small cell lung cancer (squamous)	Must have completed exposure to at least, in any order and number of LOT, 1 LOT containing carboplatin or cisplatin and gemcitabine or docetaxel or paclitaxel or paclitaxel protein-bound, and a checkpoint inhibitor (pembrolizumab, nivolumab, atezolizumab or durvalumab) at any time, including before advanced diagnosis date
Ovarian cancer	Must have completed exposure to ≥3 LOT, in any order, combination, and number of lines of therapy, which included 1 LOT containing carboplatin or cisplatin, and two

	additional LOT, with each line required to include exposure to ≥1 of the following: carboplatin or cisplatin (repeat use in a subsequent line would count as an additional line), liposomal doxorubicin, gemcitabine, topotecan, vinorelbine, etoposide, capecitabine, paclitaxel, or docetaxel
Pancreatic cancer	Must have completed exposure to ≥1 LOT and have been exposed, in any order, combination, and number of LOT, to a fluoropyrimidine-based (5-FU or capecitabine) LOT and a gemcitabine-based LOT<br>Patients may have received gemcitabine and a fluoropyrimidine within 1 LOT (eg, gemcitabine-capecitabine)
Prostate cancer	Must have completed exposure to ≥1 LOT and have been exposed to goserelin, histrelin, leuprolide, or triptorelin; docetaxel or cabazitaxel; and abiraterone, enzalutamide, apalutamide or darolutamide, in any order, combination and number of LOT, at any time, including before metastatic diagnosis date<br>Upon meeting exposure requirements, designation of last SOC line was determined by exposure to docetaxel or cabazitaxel; and abiraterone, enzalutamide, apalutamide or darolutamide
Enhanced pan-tumor CGDB	
Cervical cancer	Must have completed exposure to ≥1 LOT and have been exposed to ≥1 of the following: carboplatin, cisplatin or topotecan
High-grade gliomas (including glioblastoma)	Must have completed exposure to temozolomide and radiotherapy or 1 prior line of systemic cancer therapy (if no exposure to temozolomide was documented)[c]

Anal adenocarcinoma	Must have completed exposure to 1 TB and exposure (in any order, combination and number of TBs) to all of the following: oxaliplatin, irinotecan, and (fluorouracil or capecitabine)
Low-grade gliomas, salivary gland cancer, soft tissue sarcoma, gastrointestinal stromal tumor, malignant small round cell tumor, adenoid cystic carcinoma, basal cell carcinoma, duodenal cancer, gallbladder carcinoma, germ cell tumor, parathyroid carcinoma, testicular cancer, thymic cancer, carcinoma of unknown primary, and all other UDM tumor types	One prior line of systemic cancer therapy[d]

Abbreviations: CGDB, clinico-genomic database; LOT, line(s) of therapy; SOC, standard of care; TB, treatment block; UDM, Universal Data Model (carcinoma: cervical cancer, anal, gallbladder, small intestine (duodenal, jejunum, ileum), and head and neck; occult/unknown primary; sarcoma: unspecified/other, soft tissue sarcoma, and bone [unspecified/other]).

[a]Prespecified following clinical guidance.

[b]Requirements applied in the advanced/metastatic setting unless otherwise noted.

[c]Flatiron developed definitions of advanced/metastatic disease for disease-specific CGDBs and for diseases within the enhanced pan-tumor CGDB.

[d]Defined as the first LOT or TB given on or after the advanced/metastatic diagnosis date.
