# Supplementary material for: A Real-World Study of Patients with FGFR-Altered Advanced Solid Tumors after Exhaustion of Standard-of-Care Treatment
Source: Cancer Res Commun. 2026 Oct 20;6(10):2451–62. doi: 10.1158/2767-9764.CRC-26-0233 (PMC13680822; doi:10.1158/2767-9764.CRC-26-0233)
Supplement: Supplementary file 2 [file crc-26-0233_supplementary_table_s2_suppst2.pdf]

Supplementary table 2: Tumor types studied in the RAGNAR trial and real-world study

RAGNAR trial	Real-world study
• Cholangiocarcinoma<br>• High-grade glioma<br>• Breast cancer<br>• Pancreatic cancer<br>• Squamous non-small cell lung cancer<br>• Non-squamous non-small cell lung cancer<br>• Colorectal cancer<br>• Endometrial cancer<br>• Gastric cancer<br>• Esophageal cancer<br>• Squamous cell head and neck cancers<br>• Ovarian cancer<br>• Prostate cancer<br>• Low-grade glioma<br>• Cervical cancer<br>• Carcinoma of unknown primary<br>• Salivary gland cancer<br>• Soft tissue sarcoma<br>• Gastrointestinal stromal tumor<br>• Adenoid cystic carcinoma<br>• Anal adenocarcinoma	Disease-specific CGDB<br>• Advanced cholangiocarcinoma<br>• Breast cancer<br>• Pancreatic cancer<br>• Non-small cell lung cancer<br>○ Squamous<br>○ Non-squamous<br>• Colorectal cancer<br>• Advanced endometrial cancer<br>• Gastric/esophageal/ gastroesophageal<br>• Squamous cell head and neck cancers<br>• Ovarian cancer<br>• Metastatic prostate cancer<br>Enhanced pan-tumor CGDB<br>• Glioma, including high-grade and low-grade<br>• Carcinoma Gynecologic: cervical<br>• Carcinoma of unknown primary<br>• Carcinoma: head and neck, including salivary gland cancer<br>• Sarcoma: soft tissue sarcoma, including gastrointestinal stromal tumor and unspecified/other

• Gallbladder carcinoma<br>• Basel cell carcinoma<br>• Duodenal cancer<br>• Germ cell tumor<br>• Testicular cancer<br>• Parathyroid carcinoma<br>• Thymic cancer<br>• Malignant small round cell tumor<br>• Mesothelioma<br>• Thyroid carcinoma<br>• Conjunctival epidermoid carcinoma	• Sarcoma: bone<br>• Sarcoma: unspecified/other<br>• Adenoid cystic carcinoma<br>• Carcinoma gastrointestinal: anal, including adenocarcinoma<br>• Carcinoma: gallbladder<br>• Carcinoma skin: basal cell carcinoma<br>• Carcinoma: small intestine, including duodenal carcinoma<br>• Germ cell tumor, including testicular<br>• Carcinoma: parathyroid carcinoma<br>• Carcinoma: thymic cancer<br>• Parathyroid cancer

Abbreviation: CGDB, Clinico-genomic database.select
