# Supplementary material for: A Real-World Study of Patients with FGFR-Altered Advanced Solid Tumors after Exhaustion of Standard-of-Care Treatment
Source: Cancer Res Commun. 2026 Oct 20;6(10):2451–62. doi: 10.1158/2767-9764.CRC-26-0233 (PMC13680822; doi:10.1158/2767-9764.CRC-26-0233)
Supplement: Supplementary file 3 [file crc-26-0233_supplementary_table_s3_suppst3.pdf]

Supplementary table 3: Fibroblast growth factor receptor valine gatekeeper and resistance alterations

FGFR1	FGFR2	FGFR3	FGFR4
V561X [a]	V564X [a]	V555X [a]	V550X [a, b]
N546X	N549X [a]	N540X	N535X
			V510X [b]

Abbreviation: FGFR, fibroblast growth factor receptor.

[a]X is any amino acid resulting from a missense alteration.

[b]FGFR4 V550X and V510X are the same alteration reported in different transcripts.
