# Supplementary material for: A Real-World Study of Patients with FGFR-Altered Advanced Solid Tumors after Exhaustion of Standard-of-Care Treatment
Source: Cancer Res Commun. 2026 Oct 20;6(10):2451–62. doi: 10.1158/2767-9764.CRC-26-0233 (PMC13680822; doi:10.1158/2767-9764.CRC-26-0233)
Supplement: Supplementary file 4 [file crc-26-0233_supplementary_table_s4_suppst4.pdf]

Supplementary table 4. Real-world systemic therapy patterns by tumor type

	Overall N=230	Breast cancer n=17	Cholangio-carcinoma n=84	Endometrial cancer n=12	Gastric esophageal and gastroesophageal cancer n=12	Glioma n=20	Non-small cell lung cancer n=20
First-line therapy in the advanced/metastatic setting to standard-of-care exhaustion, n (%)[a]							
Eligible patients[b]	161 (70.0)	17 (100.0)	84 (100.0)	12 (100.0)	12 (100.0)	0 (0.0)	20 (100.0)
Alpha beta emitter	1 (0.6)	0 (0.0)	0 (0.0)	0 (0.0)	0 (0.0)	--	0 (0.0)
Antibody-conjugate	2 (1.2)	2 (11.8)	0 (0.0)	0 (0.0)	0 (0.0)	--	0 (0.0)
Chemotherapy	161 (100.0)	17 (100.0)	84 (100.0)	12 (100.0)	12 (100.0)	--	20 (100.0)
Clinical study drug	9 (5.6)	3 (17.6)	3 (3.6)	0 (0.0)	0 (0.0)	--	1 (5.0)
Hormonal therapy	15 (9.3)	11 (64.7)	0 (0.0)	2 (16.7)	0 (0.0)	--	0 (0.0)
Immunotherapy	28 (17.4)	2 (11.8)	0 (0.0)	2 (16.7)	0 (0.0)	--	20 (100.0)
Targeted/biologic[c]	19 (11.8)	1 (5.9)	1 (1.2)	1 (8.3)	8 (66.7)	--	1 (5.0)
Targeted/non-biologic[d]	11 (6.8)	7 (41.2)	1 (1.2)	1 (8.3)	0 (0.0)	--	1 (5.0)
Best supportive care to death, n (%)[a]							
Eligible patients[b]	161 (70.0)	17 (100.0)	84 (100.0)	12 (100.0)	12 (100.0)	0 (0.0)	20 (100.0)
Alpha beta emitter	0 (0.0)	0 (0.0)	0 (0.0)	0 (0.0)	0 (0.0)	--	0 (0.0)
Antibody-conjugate	3 (1.9)	3 (17.6)	0 (0.0)	0 (0.0)	0 (0.0)	--	0 (0.0)
Chemotherapy	105 (65.2)	12 (70.6)	60 (71.4)	5 (41.7)	6 (50.0)	--	12 (60.0)
Clinical study drug	25 (15.5)	0 (0.0)	19 (22.6)	2 (16.7)	2 (16.7)	--	1 (5.0)
Hormonal therapy	9 (5.6)	5 (29.4)	0 (0.0)	4 (33.3)	0 (0.0)	--	0 (0.0)
Immunotherapy	26 (16.1)	3 (17.6)	6 (7.1)	6 (50.0)	5 (41.7)	--	6 (30.0)
Targeted/biologic[c]	15 (9.3)	1 (5.9)	3 (3.6)	1 (8.3)	4 (33.3)	--	5 (25.0)

Targeted/non-biologic[d]	35 (21.7)	6 (35.3)	22 (26.2)	3 (25.0)	2 (16.7)	--	1 (5.0)

[a]Not mutually exclusive; patients could have been exposed to multiple therapy types, within the same line or across multiple lines.

[b]Only patients in the Clinico-genomic database were included in systemic therapy categorization because of the exploratory nature of treatment blocks in Flatiron Health data.

[c]Bevacizumab, bevacizumab-awwb, bevacizumab-bvzr, cetuximab, olaratumab, panitumumab, pertuzumab, ramucirumab, trastuzumab, trastuzumab-anns, or trastuzumab-dkst.

[d]Abemaciclib, afatinib, alpelisib, avapritinib, dabrafenib, dasatinib, erdafitinib, erlotinib, everolimus, lapatinib, lenvatinib, olaparib, osimertinib, palbociclib, pazopanib, pemigatinib, regorafenib, ripretinib, sorafenib, sunitinib, or temsirolimus.
