# Supplementary material for: A Real-World Study of Patients with FGFR-Altered Advanced Solid Tumors after Exhaustion of Standard-of-Care Treatment
Source: Cancer Res Commun. 2026 Oct 20;6(10):2451–62. doi: 10.1158/2767-9764.CRC-26-0233 (PMC13680822; doi:10.1158/2767-9764.CRC-26-0233)
Supplement: Supplementary file 5 [file crc-26-0233_supplementary_table_s5_suppst5.pdf]

Supplementary table 5. Differences in study conduct between the real-world study and the RAGNAR clinical trial

	Real-world study	RAGNAR
Design	Retrospective, non-interventional, observational, cohort study involved secondary analysis of data collected during routine medical care	International, single-treatment arm, phase 2 study
Delayed entry	Included	Not included
Index date	SOC exhaustion	Receipt of erdafitinib
Age requirement	≥12 years	≥18 years
Geography	United States	Asia, Europe, Oceania, South America, and the United States
Disease	*FGFR*alt advanced or metastatic solid tumors (types in RAGNAR) with evidence of SOC exhaustion	Histological demonstration of an unresectable, locally advanced, or metastatic solid tumor malignancy harboring a predefined *FGFR*1–4 mutation or fusion with an intact kinase domain, excluding *FGFR* gatekeeper and resistance alterations; measurable disease according to RECIST v1.1 or RANO criteria for primary brain tumors; disease progression on ≥1

		prior line of systemic therapy and no alternative standard therapy
ECOG-PS score	Any	0-1<br>(Lansky score ≥80 for patients ≥12 to <16 years, and Karnofsky score ≥80 for patients ≥16 to <18 years)
Key exclusions	Resistance mutation<br>Prior FGFR inhibitor treatment	FGFR gatekeeper or resistance alterations<br>Prior FGFR inhibitor treatment

Abbreviations: ECOG-PS, Eastern Cooperative Oncology Group Performance Status; FGFR, fibroblast growth factor receptor; RANO, Response Assessment in Neuro-Oncology; RECIST, Response Evaluation Criteria in Solid Tumors.
